# Progress on International Health Regulations (2005) core capacities in WHO’s Western Pacific Region

**DOI:** 10.5365/wpsar.2025.16.3.1245

**Published:** 2025-07-04

**Authors:** Kai Xiao, Qiu Yi Khut, Phuong Nam Nguyen, Ariuntuya Ochirpurev, Sean T Casey, Jessica Kayamori Lopes, Gina Samaan

**Affiliations:** a WHO Health Emergencies Programme, World Health Organization Regional Office for the Western Pacific, Manila, Philippines.

## Abstract

The International Health Regulations (2005; IHR) are a legally binding instrument for the 196 States Parties, including the 194 Member States of the World Health Organization (WHO), requiring them to build and maintain capacities across critical domains to prevent, detect and respond to public health threats. In an analysis of 15 IHR (2005) core capacity scores reported by States Parties in WHO’s Western Pacific Region from 2021 to 2023, average regional scores increased from 68% in 2021 to 72% in 2022, then declined to 66% in 2023. Seven States Parties maintained consistently strong scores (≥ 85%), whereas nine exhibited fluctuations of at least 10 percentage points. Categorizing States Parties into three groups based on geographical and economic characteristics highlighted that core capacities such as financing, food safety and the control of zoonotic diseases were areas requiring additional capacity-building, particularly among Pacific Island States Parties. Low- and middle-income States Parties also reported notable gaps in financing and infection prevention and control. These findings underscore the need to strengthen national coordination and accountability mechanisms. The strategic establishment or designation of a National IHR Authority – a key amendment introduced in the 2024 revision of the IHR – has the potential to enhance implementation by ensuring institutional leadership, fostering multisectoral collaboration and facilitating resource mobilization. However, national efforts alone may not be sufficient. Regional coordination will enhance political commitment and promote coordinated action, thereby strengthening preparedness and response capacities across diverse contexts and supporting more effective implementation of the IHR (2005).

The International Health Regulations (2005; IHR) constitute a legally binding international instrument for 196 States Parties, which include all 194 Member States of the World Health Organization (WHO) as of 1 May 2025. States Parties are obligated to establish, strengthen and maintain the necessary core health capacities across sectors to ensure the rapid detection and timely reporting of and effective responses to public health risks and emergencies, thereby contributing to global health security.*1*

Since 2005, States Parties in WHO’s Western Pacific Region, which comprises 27 Member States as of 1 May 2025, have significantly enhanced their IHR (2005) core capacities, including in surveillance, response, risk communication and laboratory systems, thus strengthening public health emergency preparedness and response, as outlined in Annex 1 of the IHR (2005).*2* However, the COVID-19 pandemic revealed vulnerabilities in global health systems, including gaps in preparedness, delays in reporting and insufficient coordination across relevant sectors and borders.*3*-*6* These challenges underscore the need to further strengthen core capacities and establish more robust mechanisms for multisectoral coordination to secure full implementation. In response, Member States commenced a process in January 2022 to amend the IHR (2005) to address these deficiencies.*7*, *8*

From 2022 to 2024, the Member State-led Working Group on Amendments to the International Health Regulations (2005) reviewed more than 300 proposed changes to the Regulations in light of experiences during the COVID-19 pandemic. After 2 years of negotiations, a set of amendments was adopted by the Seventy-seventh World Health Assembly in June 2024.*9* The amendments focus on enhanced coordination, capacity-building and rapid response mechanisms across all levels of the health security architecture.*7* These amendments aim to strengthen global health security by improving coordination, building core capacities and enabling timely responses to health threats. By meeting these obligations, States Parties are expected to contribute to preventing and mitigating the international spread of diseases.

A key amendment to the IHR (2005) requires the creation of a National IHR Authority (NIA) – that is, a national-level entity designated or established by the State Party to coordinate the implementation of the Regulations within the jurisdiction of the State Party.*10* The NIA is to be responsible for overseeing and ensuring the effective implementation of the IHR (2005). Strong multisectoral coordination is needed to effectively implement core capacities at the human–animal–environment interface; to ensure financial systems can reliably fund prevention, preparedness, response and recovery activities; to manage and reduce the risk of chemical, radiation and food safety incidents; and to maintain whole-of-government and whole-of-society coordination and policies for efficient responses to public health emergencies. These considerations highlight that strengthening core capacities requires not only technical enhancements but also strong political commitment and effective collaboration across multiple sectors.*11*

Importantly, the responsibility of the NIA is different from that of the National IHR Focal Point (NFP). NIAs will be mandated to drive policies, resource allocation and multisectoral engagement, while NFPs focus primarily on communication between WHO and States Parties. Operational NFPs are intended to ensure timely and continual communication with WHO and relevant stakeholders, and aim to ensure that health security information is conveyed accurately and promptly, including notifications, verifications and reports.*12* This precise and timely exchange of information is crucial for ensuring the early detection of and implementing effective responses to public health risks and emergencies, and is in itself a core capacity.*13*

States Parties are expected to establish and maintain the core capacities required under the Regulations. States Parties use the State Party Self-Assessment Annual Reporting (SPAR) tool to systematically evaluate their progress on implementing IHR (2005) core capacities, which they are legally obligated to do under Article 54 of the Regulations.*14* For the 15 capacities in the SPAR tool, States Parties rate their level on a scale of 1–5, with Level 1 indicating limited or no capacity and Level 5 representing advanced or sustained capacity. States Parties submit performance scores, with each level being associated with an approximate percentage, ranging from 0% to 100%, using a standardized methodology. Each indicator assesses specific technical areas, such as surveillance, laboratory capacities and systems, risk communication and community engagement, and financing. Monitoring SPAR results can help to identify gaps and prioritize capacity-building efforts.*15*

This paper analyses the self-reported IHR (2005) core capacities of 27 States Parties in WHO’s Western Pacific Region, based on their SPAR submissions. The findings will help States Parties to identify priorities for capacity-strengthening and priorities for implementing IHR (2005) amendments, including designating or establishing a NIA.

## Methods

States Parties use the SPAR tool, updated in 2021, to fulfil their annual reporting obligations under the IHR (2005).*16* For this analysis, scores of States Parties in the Western Pacific Region from 2021 to 2023 were obtained from the electronic SPAR platform, which is publicly available. To analyse core capacities in the Region, the average SPAR score for each of the 15 indicators was calculated, rounded to the nearest whole number and colour coded for the years 2021, 2022 and 2023. The colour coding represents the level of implementation of each core capacity, and higher scores indicate greater capacity, based on self-reporting. The colour scheme is: red (0–20), orange (21–40), yellow (41–60), light green (61–80), dark green (81–100) and grey for unreported.

For the analysis, States Parties were categorized into three groups – high-income, low- and middle-income, and Pacific Island – based on geographical and economic characteristics, using 2023 World Bank classifications.*17* A radar chart was used to visualize the overall score for each core capacity (abbreviated as C1–C15). States Parties with missing data were excluded from the analysis.

## Results

A total of 26 States Parties reported on their core capacities in 2023, compared with 19 in 2022 and 22 in 2021 (**Table 1**). All 27 Member States submitted reports at least once during the 3-year period, with 19 reporting every year. The regional average score increased from 68% in 2021 when 22 States Parties reported to 72% in 2022 when 19 reported, and then declined to 66% in 2023 when 26 reported (**Table 1**).

**Table 1:** Average score (%) for International Health Regulations (2005) core capacities for States Parties, measured by the Self-Assessment Annual Report tool, WHO Western Pacific Region, 2021–2023^a^

State Party (*n* = 27)	Year			-	-
	2021	2022	2023		
**Australia**	**88**	**89**	**89**
**Brunei Darussalam**			**71**
**Cambodia**	**57**	**60**	**68**
**China**	**94**	**93**	**94**
**Cook Islands**	**59**	**71**	**68**
**Fiji**	**54**	**48**	**55**
**Japan**	**98**	**99**	**99**
**Kiribati**	**64**		**40**
**Lao People's Democratic Republic**	**51**	**53**	**55**
**Malaysia**	**85**	**89**	**89**
**Marshall Islands**			**53**
**Federated States of Micronesia, Federated States of**	**43**	**53**	**53**
**Mongolia**	**78**	**72**	**66**
**Nauru**			**38**
**New Zealand**	**85**	**85**	**85**
**Niue**		**69**	**50**
**Palau**	**47**		**57**	**Scores**
**Papua New Guinea**			**42**	**81–100**
**Philippines**	**60**	**67**	**64**	**-**
**Republic of Korea**	**95**	**99**	**99**	**61–80**
**Samoa**	**49**	**51**	**46**	**-**
**Singapore**	**94**	**94**	**94**	**41–60**
**Solomon Islands**	**51**		**51**	**-**
**Tonga**	**55**	**70**	**70**	**21–40**
**Tuvalu**	**61**			**-**
**Vanuatu**	**74**	**54**	**56**	**0–20**
**Viet Nam**	**64**	**52**	**54**	**-**
**Regional average**	**68**	**72**	**66**	**Unreported**

Seven States Parties (Australia, China, Japan, Malaysia, New Zealand, Republic of Korea and Singapore) maintained strong and stable scores, consistently exceeding 85%. Nine States Parties (Cambodia, Cook Islands, Kiribati, Federated States of Micronesia, Niue, Palau, Tonga, Vanuatu and Viet Nam) exhibited large fluctuations in their scores, of 10 points or more. One State Party (Mongolia) reported a slight decline, and two (Cambodia and Lao People's Democratic Republic) reported steady increases in core capacities across the years.

**Fig. 1:**
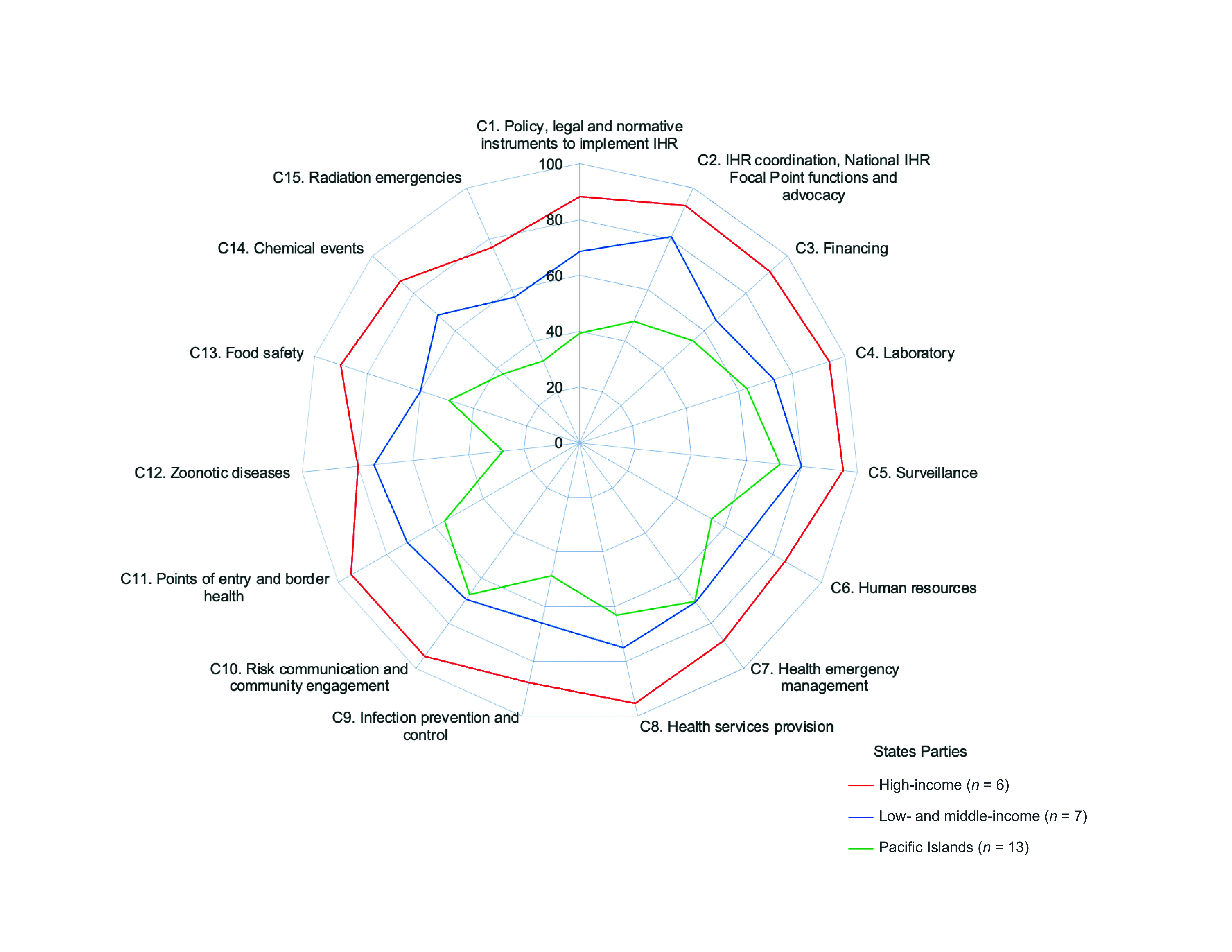
Average score of States Parties (N = 26) on specific International Health Regulations (2005) core capacity, by income or geographical area, WHO Western Pacific Region, 2023^a^

Among 26 of the 27 States Parties that reported in 2023 (**Fig. 1**), good capacity (≥ 60% score) was reported for laboratory (C4), surveillance (C5), health emergency management (C7), health services provision (C8) and risk communication and community engagement (C10). The most significant gaps in core capacities were reported for zoonotic diseases (C12), food safety (C13), chemical events (C14) and radiation emergencies (C15). Scores varied across income and geographical groupings, with high-income States Parties generally posting higher overall scores, while Pacific Island States Parties demonstrated more limited capacity across several domains, and low- and middle-income States Parties reported lower capacities in financing (C3) and infection prevention and control (C9).

[**insert** Figure 1]

## Discussion

The status of IHR (2005) core capacities among States Parties in WHO’s Western Pacific Region reflects diversity in national systems, resources and contexts. High-income countries report consistently high scores, while many Pacific Island and low- and middle-income countries face challenges due to limited human resources, geographical dispersion and reliance on external support for key public health functions. These contextual differences influence not only the development of national capacities but also the comparability of progress across the Region. As a result, regional average scores can be influenced by which States Parties report. One possible reason for the decrease in the average score from 2022 to 2023 is the increase in reporting by some lower-scoring Pacific Island countries, which may have affected the overall regional average. While some States Parties have made notable progress in areas such as surveillance, laboratory services and emergency management, all States Parties have opportunities to further strengthen specific domains, particularly food safety, zoonotic disease control and sustainable financing. Sustained investment and coordination are critical for ensuring that all States Parties can effectively prevent, detect and respond to public health threats.

Joint External Evaluations (JEEs), another key tool within the IHR (2005) Monitoring and Evaluation Framework, complement SPAR by providing external, qualitative assessments that help identify strengths and priorities. In the Western Pacific Region, JEEs conducted in several countries have offered important context to enable better interpretation of SPAR findings, thus providing additional insights for capacity-strengthening, especially when self-assessments may overlook operational challenges. For instance, States Parties in the Pacific Island countries have highlighted persistent workforce shortages, limited surge capacity and the critical need for multisectoral coordination mechanisms to rapidly mobilize external support in response to chemical or radiation emergencies or other acute public health hazards. In particular, geographical dispersion continues to pose significant challenges for Pacific Island States Parties.*18*-*24*

Meaningful progress has been made by States Parties in strengthening core capacities. For example, by establishing and reinforcing emergency medical teams (EMTs), States Parties have bolstered their ability to rapidly respond to outbreaks and disasters, thus strengthening the IHR (2005) core capacities critical for effective health emergency management and international collaboration. Since the inception of the EMT Initiative in 2010 following the devastating Haiti earthquake, 16 of the 53 WHO-classified EMTs (31%) that have been established are in the Western Pacific Region.*25* Alongside the EMTs classified for international response, nearly every Member State in the Region has established a national EMT or is in the process of doing so. This means that nearly all States Parties in the Region have domestic EMTs ready to provide surge assistance to others in times of crisis. In recent years, EMTs from the Western Pacific Region have deployed to provide rapid clinical care during disasters, outbreaks and mass gathering events, and they have also helped build local capacities during joint training and simulation exercises.*26* Their presence and coordinated action facilitate knowledge transfer and enhance emergency management capacities. The measures taken to establish and reinforce EMTs demonstrate that while many States Parties have relatively small health systems, they can leverage regional solidarity and external technical assistance to address chemical, biological and radiological incidents more effectively.*27*-*32*

A similar story can be told about the regional uptake of the Global Outbreak Alert and Response Network (GOARN), with 80 of the 320 (25%) global partner institutions coming from the Western Pacific Region.*33* Nearly 90 GOARN missions were conducted in the Region during the COVID-19 pandemic, and the mechanism was more recently used to respond to measles events to bolster clinical management and infection prevention and control activities.*34* The experts deployed not only supported immediate needs but also provided training to prepare health systems for future outbreaks. Many investments in EMTs, GOARN and other surge mechanisms have been supported from within the Region, reflecting strong solidarity, alongside contributions from international partners and other countries. These mechanisms not only strengthen regional response capacities but also enable cross-border collaboration, ensuring that expertise and resources can be mobilized swiftly within and beyond the Western Pacific Region when needed.*35*

While zoonotic diseases (C12) are noted as one of the weaker core capacities across the Region, ongoing regional initiatives aim to strengthen this area through collaborative approaches. Fourteen of 27 States Parties in the Western Pacific Region have established multisectoral coordination mechanisms, integrating the human health, animal health and environmental health sectors to detect and contain zoonotic threats more efficiently.*36*, *37*

Viet Nam’s integrated response to a 2024 *Salmonella* outbreak related to banh mi demonstrated effective multisectoral coordination, ensuring rapid containment. Authorities immediately mobilized laboratories, environmental and epidemiological surveillance, and risk communication, enabling swift confirmation of the outbreak and public advisories to prevent further spread.*38* During the outbreak, the WHO International Food Safety Authorities Network (INFOSAN) played a crucial role in accelerating the exchange of information and coordinating food safety actions. While the IHR (2005) mandates international notification and management of public health risks, INFOSAN facilitates real-time technical collaboration among food safety agencies. By enabling swift data-sharing and coordinated risk mitigation, INFOSAN complemented IHR (2005) mechanisms, ensuring a timely and effective response. As of December 2024, all 27 States Parties in the Western Pacific Region have INFOSAN contact points. By engaging in the Network, States Parties can bridge capacity gaps, share critical data and coordinate timely responses to protect public health.*39* Investing in food safety has major benefits for strengthening surveillance systems, including for monitoring antimicrobial resistance and providing genomic surveillance of foodborne pathogens and resistant organisms, further adding to the value of the overall food supply chain. Continual regional efforts to improve the management of food safety incidents are critical for food safety systems, especially maintaining sustainable funding and political support.

Despite notable progress in some core capacities, many States Parties still face challenges in managing hazards that require robust, multisectoral coordination, especially chemical (C14), radiological (C15) and food safety (C13) events. The recent amendments to the IHR (2005) underscore the importance of strengthening core capacities to address evolving public health threats. Establishing a NIA provides an opportunity to further strengthen multisectoral collaboration, resource integration and international collaboration. An effectively empowered NIA can coordinate these efforts by engaging multiple stakeholders and driving both whole-of-government and whole-of-society approaches. A well resourced NIA can optimize resource allocation, streamline decision-making and foster transparent information-sharing, thereby making steady progress towards more robust implementation of the IHR (2005). In turn, this progress helps address persistent gaps in areas such as food safety, chemical and radiological preparedness, and risk communication.

Additionally, cross-border public health threats call for stronger regional coordination. Dedicated regional coordination of political, technical and operational government actors, facilitated by WHO, will strengthen political commitment, coherence and resource mobilization among Member States while enhancing global health collaboration and ensuring swift, equitable responses to crises.*40* For example, where establishing complete domestic capacity is not practical – especially for certain chemical or radiation events – States Parties may benefit from the capacities available through existing regional networks and technical support arrangements, which allow resource-limited States Parties to leverage international expertise, as needed.*41*

These considerations are especially relevant given the low likelihood but potentially high impact of certain incidents, such as chemical or radiation events, in many island settings, as well as the prohibitive costs of maintaining in-country capacities to respond to some of these lower-likelihood hazards. As a result, several Pacific Island States Parties rely on formal agreements with larger neighbours or regional hubs for technical expertise, including laboratory analyses. Such arrangements underscore the need for well–defined protocols and multisectoral mechanisms, particularly in advance of events such as chemical spills, radiation leaks or other complex hazards, to enable the rapid mobilization of external support.*42*

This regional analysis is based on self-reported data from SPAR, which may over- or underestimate actual capacities due to reporting bias or differences in reporting quality and completeness across countries.*43* It is important to note that strengthening data collection and information-sharing practices is crucial for gaining a comprehensive understanding of progress made in improving core capacities. Annual State Party reporting represents an important step in sharing knowledge and in transparency, and these can guide investments and strategic actions. Incorporating qualitative assessments, such as JEEs, alongside SPAR results can highlight the nuances of challenges and opportunities. Regular reporting through the SPAR tool, mandated under the IHR (2005), and the proactive exchange of experiences among States Parties promote transparency, can help identify best practices, can facilitate joint action to address common limitations, and can leverage resources that can be shared across borders. Overall, sustaining progress in IHR (2005) core capacities requires strong national leadership and coordination. NIAs can play a critical role in furthering the implementation of the core capacities in States Parties by aligning policies, resources and multisectoral action, and the NIAs are central to translating assessments into concrete improvements. Strengthening national systems while leveraging regional diversity and collaboration will be key to building resilient systems and enhancing collective health security.
